# Bibliography

**Published:** 1886-02

**Authors:** 


### BIBLIOGRAPHY.

### BOOKS AND PAMPHLETS RECEIVED.

*A Treatise on Nervous Diseases—Their Symptoms and Treatment*, a text-book for students and practitioners, by Samuel G. Webber, M. D., Clinical Instructor on Nervous Diseases, Harvard Medical College, etc. New York, D. Appleton & Co., 1885; pp. 415.

This work seems to have been designed for a kind of compendium of nervous diseases—as the author says, to furnish a practitioner with what he most needs to know for diagnosis and treatment of cases occurring in practice. To this end all theories and long discussions have been omitted—even contributions to the sum total of knowledge have not, in every instance, been credited; only facts, accepted facts, are stated, and stated briefly. It is not, therefore, an exhaustive treatise, as the name would imply, but a kind of summary of known facts or accepted teachings. Therofore, it is not, as the author says, a "book for the specialist," the condensation having been carried, it may seem, too far. But, says the author, a more extensive description would have required an increase in the bulk of the book, and would thereby have defeated the main object, which was to submit what is most essential for the study of nervous diseases within as small a lump as possible. It is good reading.

---

*Milk Analysis and Infant Feeding:* a practical treatise on the examination of human and cow's milk, cream, condensed milk, etc., and directions as to the diet of young infants. By Arthur V. Meigs, M. D. Cloth; pp. 102. Philadelphia: P. Blakiston Son & Co., 1885. Price, $1.

This little volume contains an amount of useful information and a number of practical suggestions as to artificial feeding of infants, in cases where, from any cause, the child is deprived of the mother's breast ; *a very important* subject, when we consider that by far the largest number of infants who die, do so from gastro-intestinal troubles, the result of *errors in diet*, and those deaths constitute about twenty-five per cent. of the whole, from all causes. It will pay any physician to avail himself of these suggestions and the information embodied in this modest little volume.

---

*Lectures on Diseases of the Nose and Throat*, delivered during the Spring Session of Jefferson Medical College, by Charles E. Sajous, M. D., Lecturer on Rhinology and Laryngology etc., etc., illustrated with one hundred chromo-lithographs from oil paintings by the author, and ninety-three engravings on wood, Philadelphia: F. A. Davis, Attorney, Publishers, 1217 Filbert street, 1885, p. 416; cloth $4.00, Leather $5.00.

The young house of Davis comes to the front with this valuable and attractive book, gotten up in the highest style of the modern bookmaker's art. It is selling rapidly, and fills a nitch in the requirements of every physicians practice. The plates are superb; each one having been drawn from photographs of the larynx and posterior nares, etc., made by Dr. Sajous' own instantaneous process, which is a marvel of ingenuity; and colored by himself in oil. One valuable feature of the book is the author's prescriptions, which he gives in detail, with an analysis of their action.

In addition to the subject proper, which is treated with a most satisfactory thoroughness and attention to detail, there is a chapter on hay fever, in which is given the Doctor's treatment, which attracted so much attention at the time it was given to the profession in separate form, on account of his singular success in the management of this intractable disease. On hay fever, as on diseases of the throat and nose, Professor Sajous is an acknowledged authority, both in Europe and America.

This handsome work is sold only by subscription, and at the extremely low price of $4 in cloth. It will constitute both an ornament and useful addition to any library.

We have received the following works, which we hope to be able to mention more at length when we have had an opportunity to examine their contents :

From D. Appleton & Co., 1, 3 and 5 Bond street, New York, a *Text-Book of Ophthalmoscopy*, by Edward G. Loring, M. D., illustrated.

From P. Blakiston Sons & Co., 1012 Walnut street, Philadelphia, a *Compend of Medical Practice*, by Hughes.

From the Secretary, J. Berrien Lindsley, Nashville, Tenn., *Second Report of the Tennessee State Board of Health.*

From Boykin, Cramer & Co., Baltimore, *A Doctor's Experience in Three Continents*, by Edward Warren-Bey, M. D., etc.

From H. Campbell & Co., 140 Nassau street., New York, *Bartley's Urinary Test Case.*

This is one of the most useful articles a physician could have; indeed, it is one of the most valuable aids to diagnosis yet devised by the chemist. By means of the contents of this little case, which can be carried in a vest pocket, the physician can, at the bedside, take the specific gravity of the urine—estimate the quantity of urine passed, the color, reaction, specific gravity, solids passed, and the presence or absence of albumen or sugar. Now the test for sugar has always been a difficult, tedious and unsatisfactory affair, and there were few druggists and fewer physicians who could do it, or even knew what constituted the test, so complicated were the steps necessary; but here in this little arrangement, all is simplified. The case contains a correct urinometer, a heavy glass test tube, serving as a urinometer and test tube, a package of litmus test papers, a pipette for convenience of handling the urine, two vials to contain test powders, and a spoon. Each bottle contains sufficient powders for fifty tests. A small hand-book of instructions, formulæ, etc., accompanies each case. Price, $2 by mail, post-paid. [This is not a book, but here is a good place to notice it.—Ed.]

---

*The Use of the Microscope in Clinical and Pathological Examinations*, by Carl Friedlander, Privat-Docent in Pathological Anatomy, at Berlin; second edition, enlarged and improved, with chromo-lithographs, Translated, by permission of the author, by Henry C. Coe, M. D., M. R. C. S. L. R. P. (London), Pathologist to The Woman's Hospital, New York. D. Appleton & Co., New York, 1, 3 and 5, Bond street, New York; 1885.

The name of the distinguished author of this work is sufficient guarantee of its worth. To those who appreciate the aid furnished by the microscope in clinical and pathological investigations, this work is invaluable; to all, it will prove interesting and instructive. The scope of the work is indicated by the following reference to the table of contents:

Chapters Nos. 1 and 2 give a description of the microscope, its accessories, objectives, Abbe's illuminating apparatus, etc.; chapter 3 embraces micro-chemistry and the re-agents, dyes, etc., used in microsocopy. There are too many good things in the book to attempt an enumeration. Chapters 4 and 5 describe other methods of preparing tissues for examination with the microscope, and how to observe living tissues, to-wit: the circulation of the blood, etc. Chapter 6, which is devoted to a description of examination of fluids, is admirable, to-wit: blood, sputa, pus, urine and exudation; the technique of preparing, staining, mounting and examining these fluids is given in this chapter. Chapter 7 is devoted to the examination of the solids of the body—tumors, etc. This valuable little volume will be found of absorbing interest to the student of pathological anatomy and microscopy. It is turned out with the usual attention to detail that characterizes all of Appleton's publications. McL.

---

YOUTH'S COMPANION.—This valuable and interesting publication, which has been before the people some fifty years, having stood the test of time and criticism likewise, grows better with age. Young persons of both sexes should have appropriate literature and plenty of it. Nothing tends to shape the character and to refine the thoughts so powerfully as one's early reading. It is a serious matter, the kind of literature parents permit their children to get hold of. We recommend the *Youth's Companion* as chaste and instructive, and advise parents to get it for their little sons and daughters. Perry Mason & Co., 41 Temple Place, Boston, proprietors.

